# A Case of McLeod’s Syndrome Presenting with Severe Decompensated Heart Failure

**DOI:** 10.14797/mdcvj.1164

**Published:** 2023-09-14

**Authors:** Hemanth K. Boppana, Samarthkumar Thakkar, Harsh P. Patel, Rody G. Bou Chaaya, Scott Feitell

**Affiliations:** 1 Rochester General Hospital, Rochester, New York, US; 2 Houston Methodist DeBakey Heart & Vascular Center, Houston, Texas, US; 3 Southern Illinois School of Medicine, Springfield, Illinois, US; 4 Sands Constellation Heart Institute, Rochester General Hospital, Rochester, New York, US

**Keywords:** McLeod’s syndrome, guideline-directed medical therapy, implantable cardioverter defibrillator, late galodinium enhancement

## Abstract

McLeod’s syndrome (MLS) is an X-linked disorder caused by mutations in the XK gene with neurological manifestations as well as cardiomyopathy. This is a case of acute exacerbation of heart failure in a 44-year-old White male with a confirmed diagnosis of MLS, which was managed with guideline-directed medical therapy and placement of an implantable cardioverter defibrillator with recovery in ejection fraction.

## Case Presentation

A 44-year-old White male presented with progressively worsening dyspnea on exertion and bilateral leg swelling. Past medical history was significant for McLeod’s syndrome (MLS) diagnosed at 6 years of age, hyperlipidemia, hypertension, and type 2 diabetes mellitus. Family history was notable for McLeod’s syndrome, premature coronary artery disease, and sudden cardiac death. Cardiac workup pursued a few months prior, due to similar complaints, revealed a left ventricular ejection fraction (LVEF) of 45% along with hypokinesis of the entire anterior wall and apical anterior wall and severe pulmonary hypertension. Angiogram findings were within normal limits, with cardiac catheterization showing mildly elevated LV end diastolic pressure.

On examination, the patient was tachypneic and tachycardic with mild bibasilar crackles and pitting edema of bilateral lower extremities without elevation in the jugular venous pressure. Laboratory investigations were significant for brain natriuretic peptide of 1,224 pg/mL, serum sodium of 128 mmol/L, and mild elevation in liver enzymes. Chest radiograph showed nonspecific patchy linear densities at bilateral lung bases, and electrocardiogram showed sinus tachycardia with nonspecific ST-T wave changes. Computed tomography chest with pulmonary embolus protocol was negative for pulmonary embolism.

**Figure 1:**
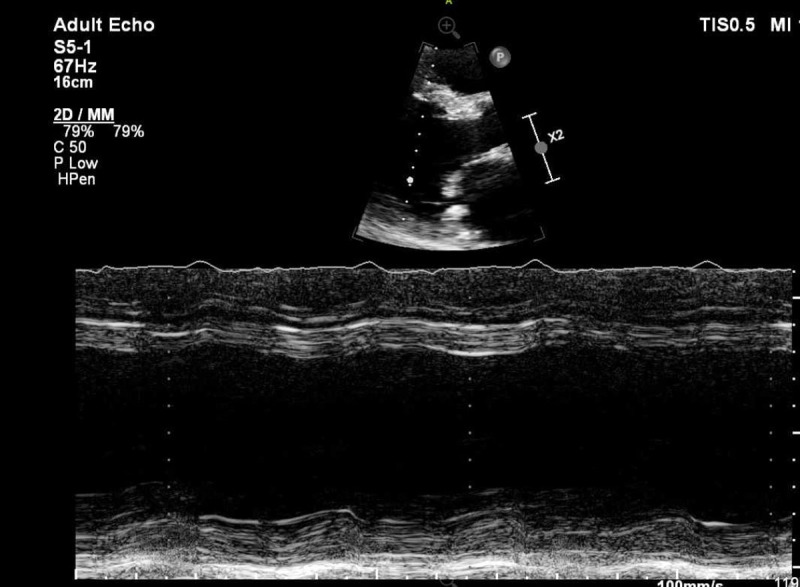
Two-dimensional and M-mode echocardiogram showing a significantly dilated left ventricle.

**Figure 2:**
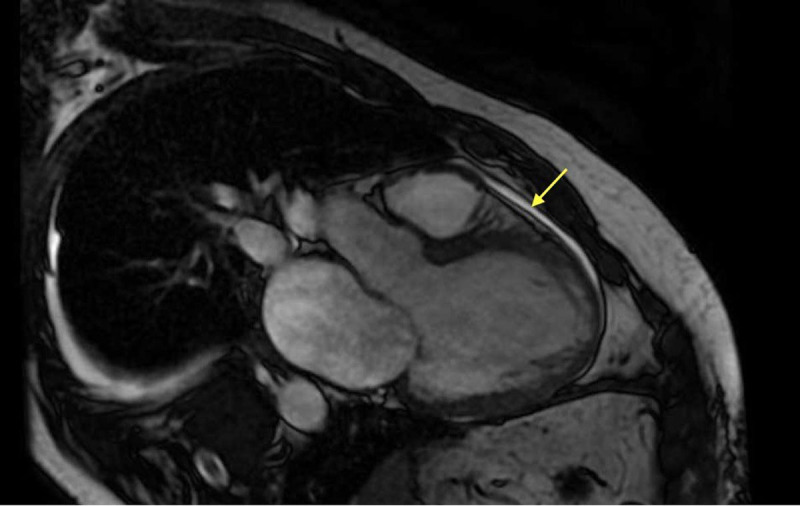
Cardiac magnetic resonance imaging with diffuse late gadolinium enhancement (yellow arrow) consistent with myocardial scarring.

Echocardiogram showed severe LV systolic dysfunction with EF of 10%, global hypokinesis and dilation of the LV (Figure 1), dilated and mildly hypokinetic right ventricle, and moderate to severe pulmonary hypertension. Hospitalization was complicated by nonsustained supraventricular tachycardia (NSVT), for which the patient underwent cardiac magnetic resonance imaging (MRI), which showed diffuse late gadolinium enhancement consistent with myocardial scarring (Figure 2). Extensive workup for cardiomyopathy was negative except for genetic sequencing of the XK gene that was positive for the McLeod phenotype.

The patient was treated for acute exacerbation of heart failure with aggressive diuresis and was started on guideline-directed medical therapy (GDMT) with digoxin, metoprolol succinate, sacubitril-valsartan, spironolactone, and dapagliflozin. Due to the presence of extensive scarring seen on cardiac MRI and recurrent runs of NSVT, the decision was made to proceed with placement of an implantable cardioverter defibrillator (ICD) due to risk of sudden cardiac death. A repeat echocardiogram a few months later showed mildly reduced LV systolic function with mild global hypokinesis and LVEF of 45% along with normal Doppler-derived pulmonary artery systolic pressures.

## Discussion

MLS is an X-linked disorder associated with a mutation in the XK gene. It manifests with hematological abnormalities including acanthocytes, neurological abnormalities with chorea, cognitive impairment, psychiatric disorders, and cardiac disease.^1^

Many cases of MLS have been described in the literature, with most patients presenting with neurological symptoms; a diagnosis of MLS was made either by identifying acanthocytes in the blood or through elevated creatine kinase levels. A 1992 literature review looked at 31 cases of MLS where it was found that movement disorders, common in MLS, develop later in the disease progression.^2^ In another study that looked at 22 males between the ages of 27 to 72, only one patient had an initial presentation with a cardiac-related manifestation of atrial fibrillation, with the majority of patients presenting with neurological manifestations of movement disorders or seizures.^3^ Of 22 total patients, 11 had cardiac involvement, with 4 diagnosed with cardiomyopathy, 5 with atrial fibrillation, and 1 with atrial flutter.^3^ Based on the data collected, the study authors concluded that patients presented with cardiac disease symptoms from the fifth decade onward, similar to our case. Here, we describe a 44-year-old male who was diagnosed with MLS at the age of 6 and had a significant family history of sudden cardiac death. He presented with symptoms of acute heart failure exacerbation and was found to have dilated cardiomyopathy (DCM). In our patient, the initial manifestation of MLS was nonischemic DCM in the absence of neurological symptoms and a normal creatine kinase level.

Cardiomyopathy and cardiac arrhythmias are common findings in MLS. Cardiac arrhythmias, especially ventricular tachycardias, are a point of concern in patients with MLS due to the presence of extensive myocardial scar tissue. A 2021 study by Quick et al. that looked at six patients with chorea-acanthocytosis and six patients with MLS showed that those with MLS had a greater extent of late gadolinium enhancement or myocardial scarring, which predisposed patients to ventricular arrhythmias.^4^ These findings are similar to our patient, who had multiple runs of NSVT along with diffuse late gadolinium enhancement on cardiac MRI that was not consistent with ischemia. Given the high risk of myocardial scarring associated with MLS, which subsequently predisposes patients to life-threatening arrhythmias and risk of sudden cardiac death, we conclude that obtaining a cardiac MRI early in the disease course along with having a low threshold for early placement of ICD is beneficial for these patients. To the best of our knowledge, there have been no studies in the literature that have investigated the timing of ICD placement in this patient population.

The standard of care for heart failure with reduced ejection fraction is the initiation of GDMT with beta-blockers, aldosterone antagonists, angiotensin converting enzyme/angiotensin receptor blockers, angiotensin receptor/neprilysin inhibitors, and SGLT-2 inhibitors in any order.^5^ We conclude that early initiation of GDMT in the management of DCM secondary to MLS is beneficial as evidenced by the improvement in LVEF in our patient. Although data on the effectiveness of GDMT in the treatment of cardiomyopathy secondary to MLS is limited at this time, it appears to be promising based on our experience.

## Conclusion

The use of GDMT for treating MLS appears to be promising as evidenced by the improvement in LVEF in our patient. In addition, we suggest obtaining a cardiac MRI in patients with MLS to evaluate for the degree of scarring, as this would guide further management. We also advocate for a low threshold for early ICD placement.
